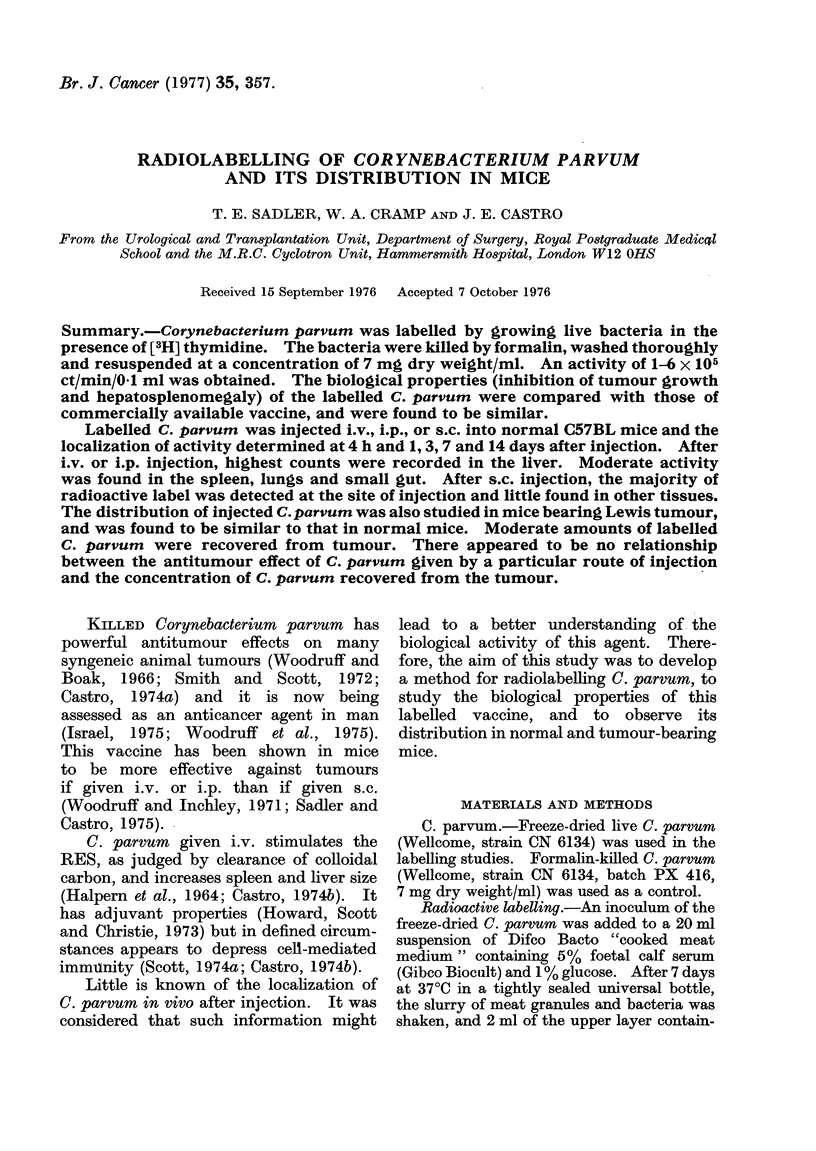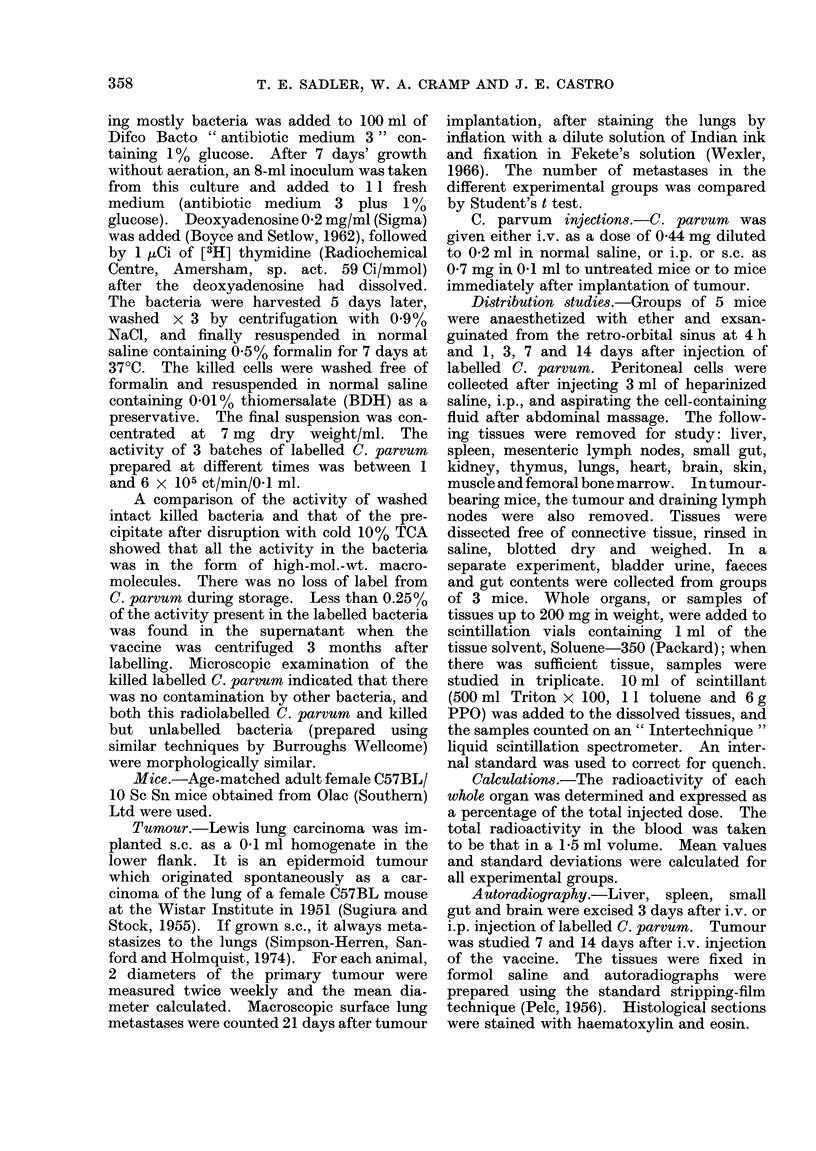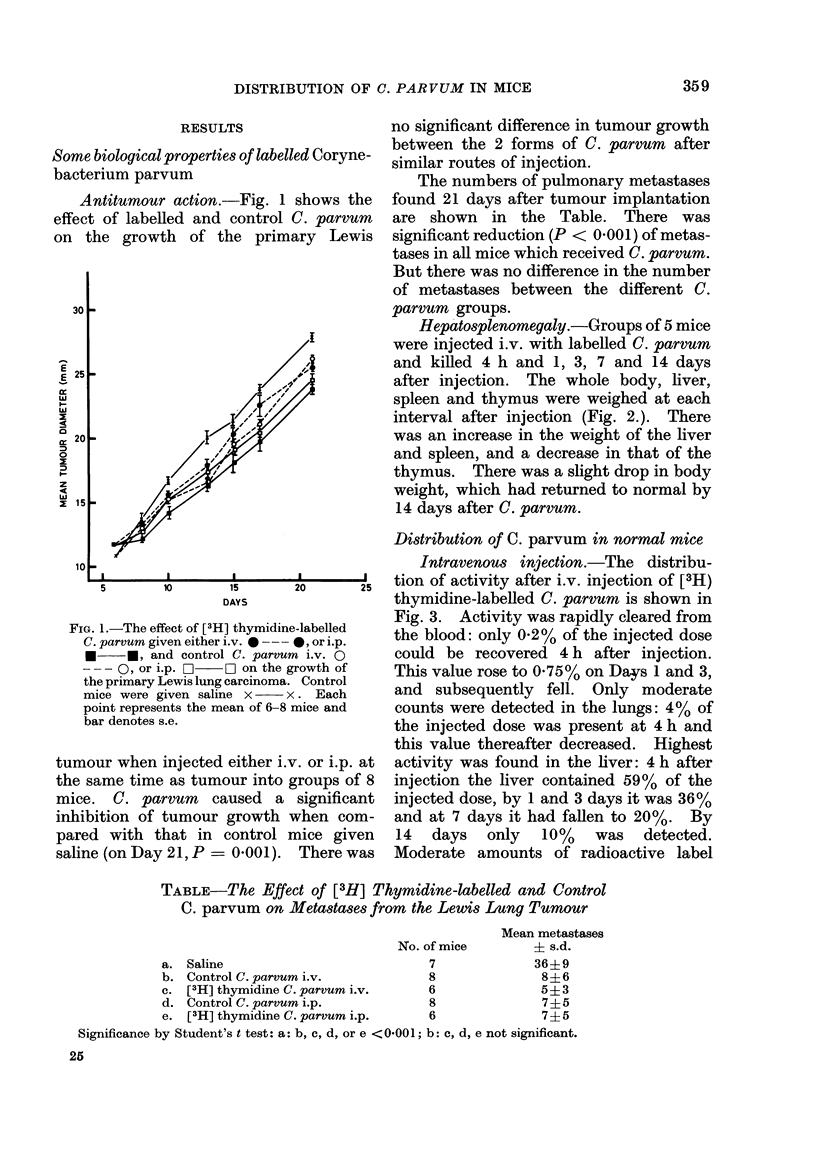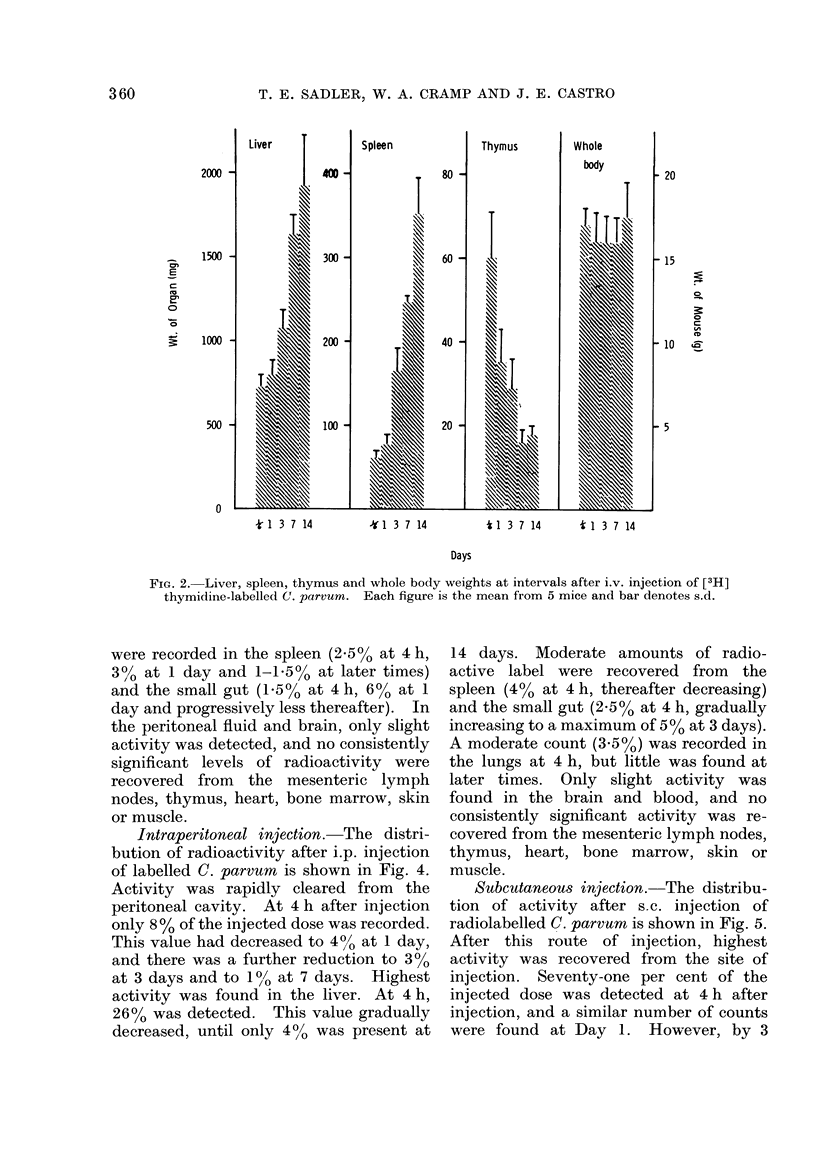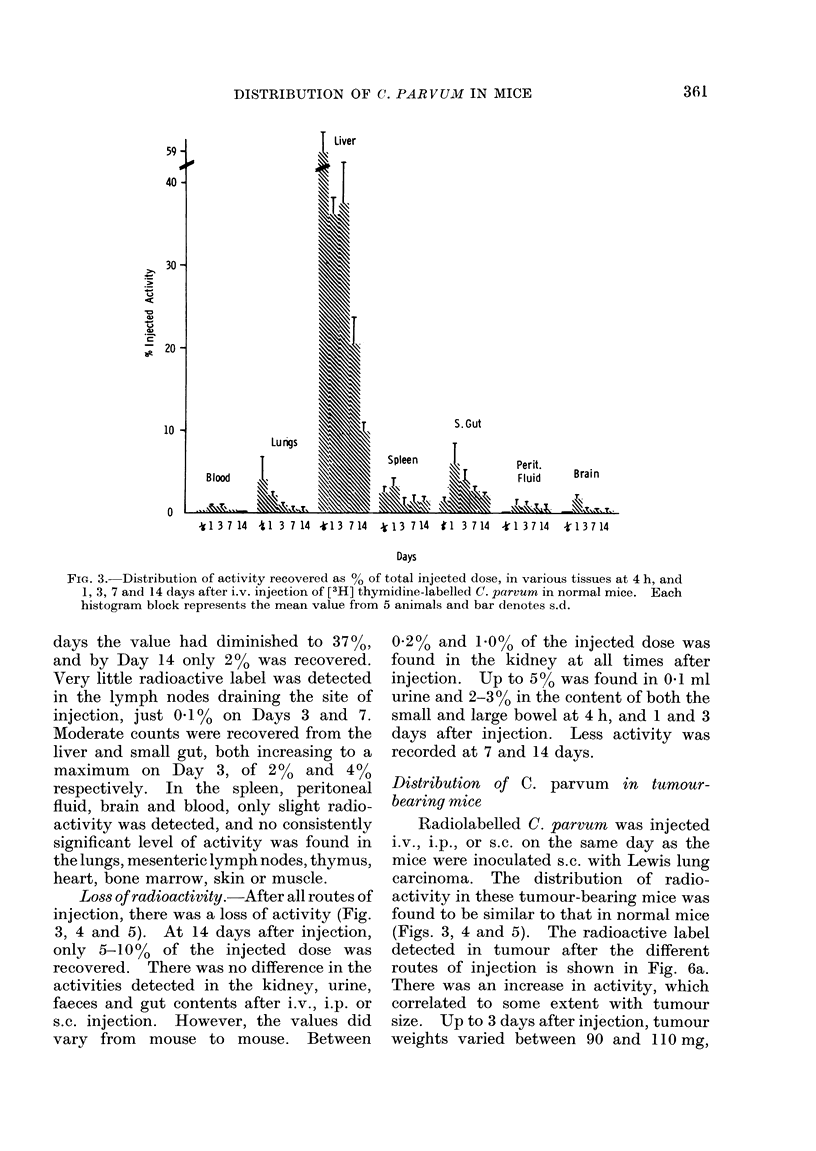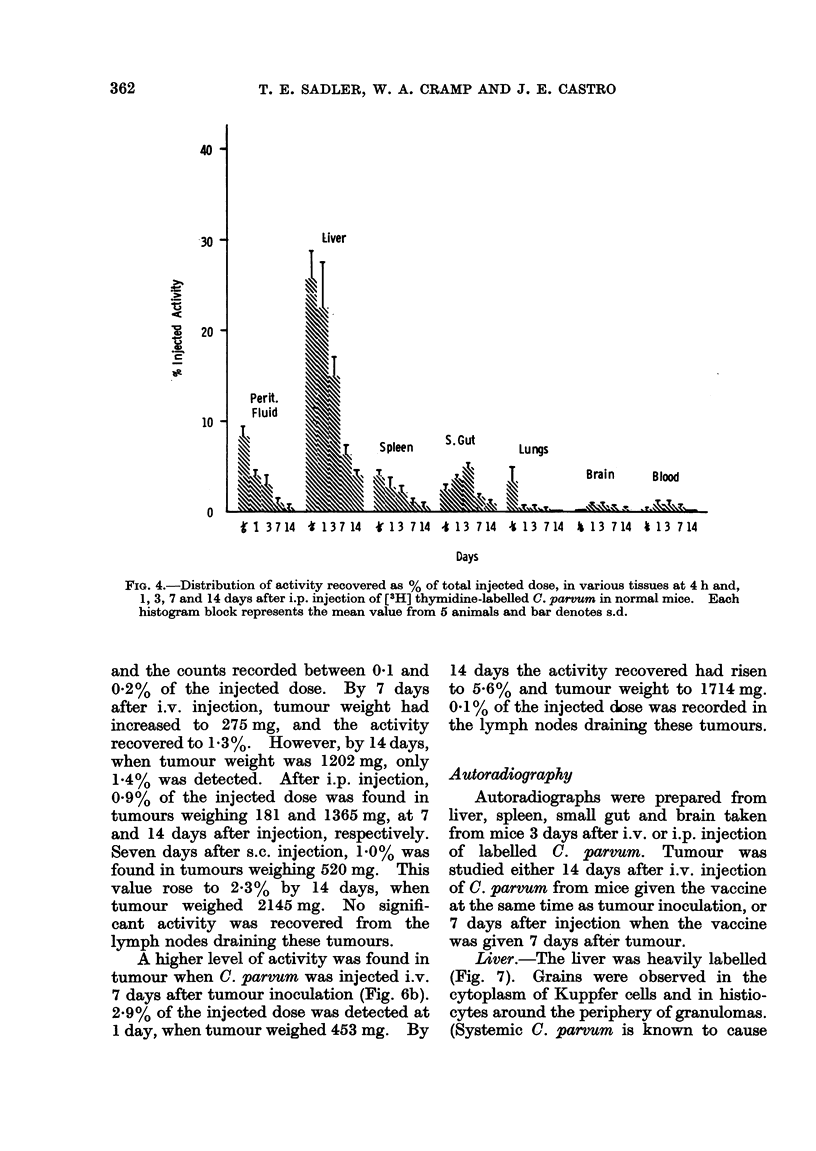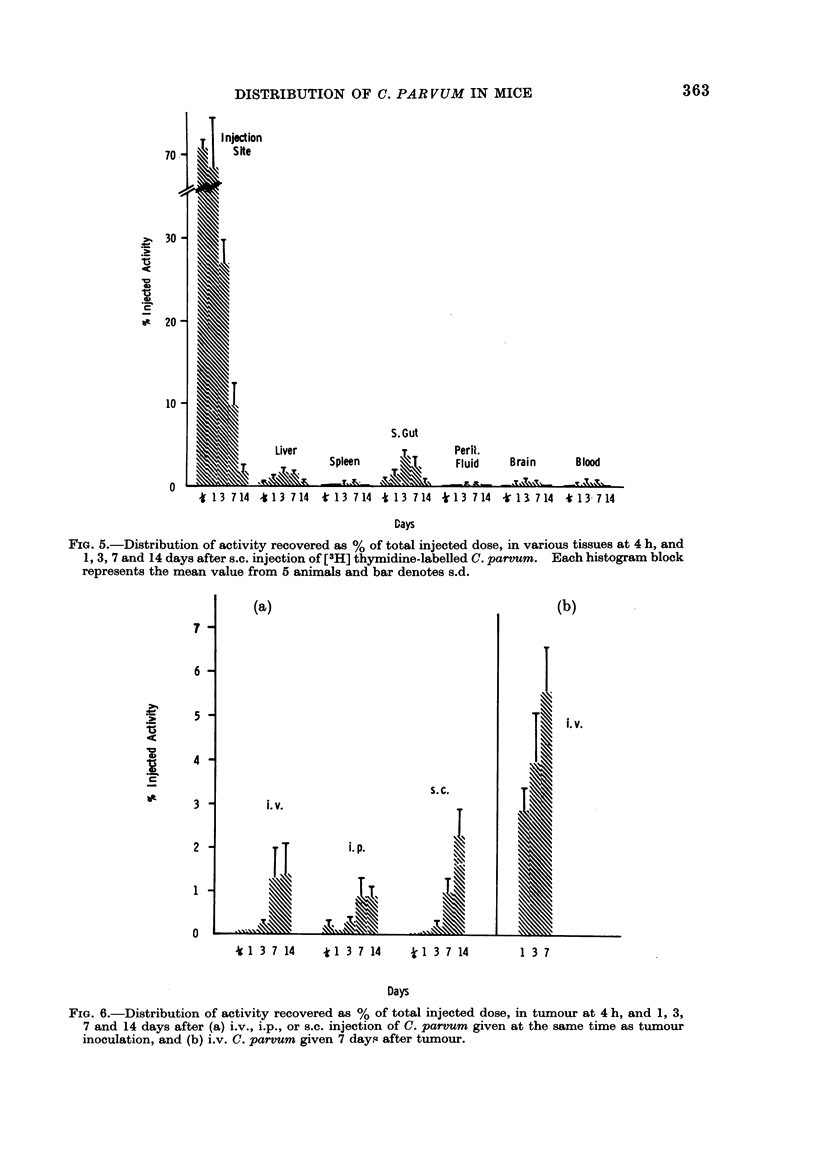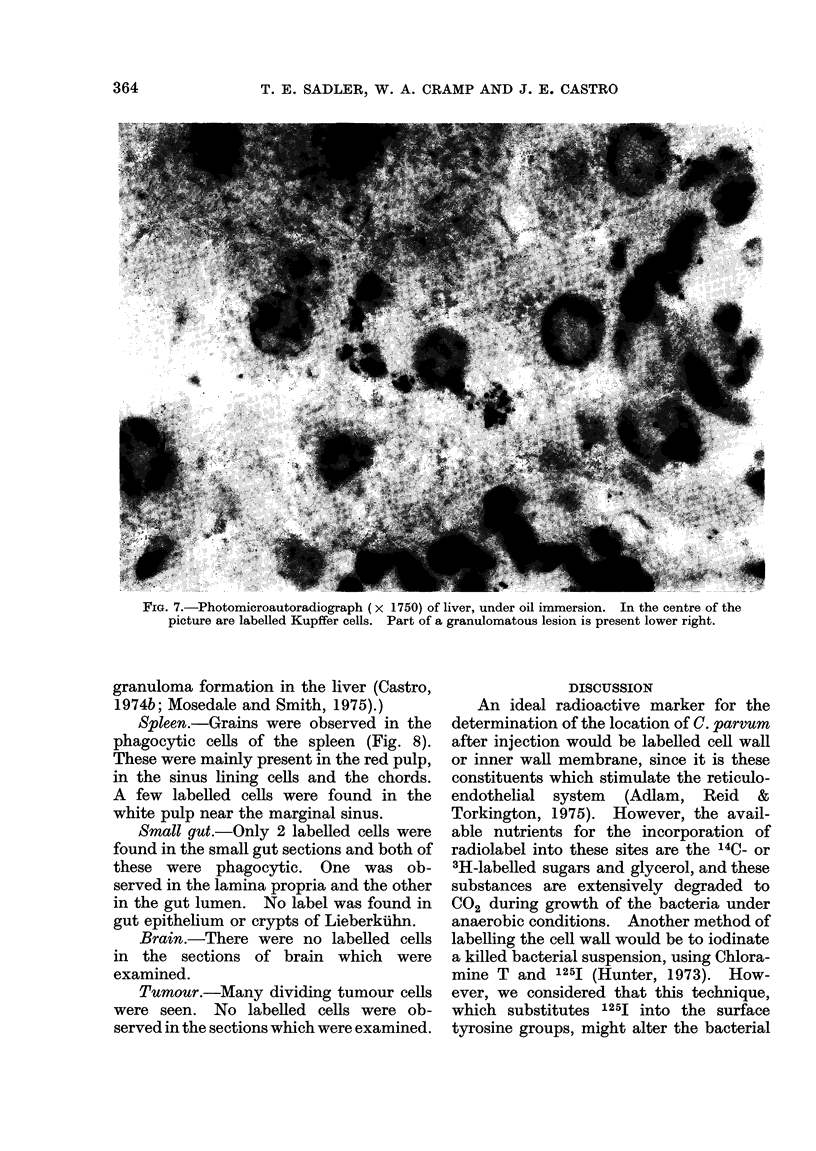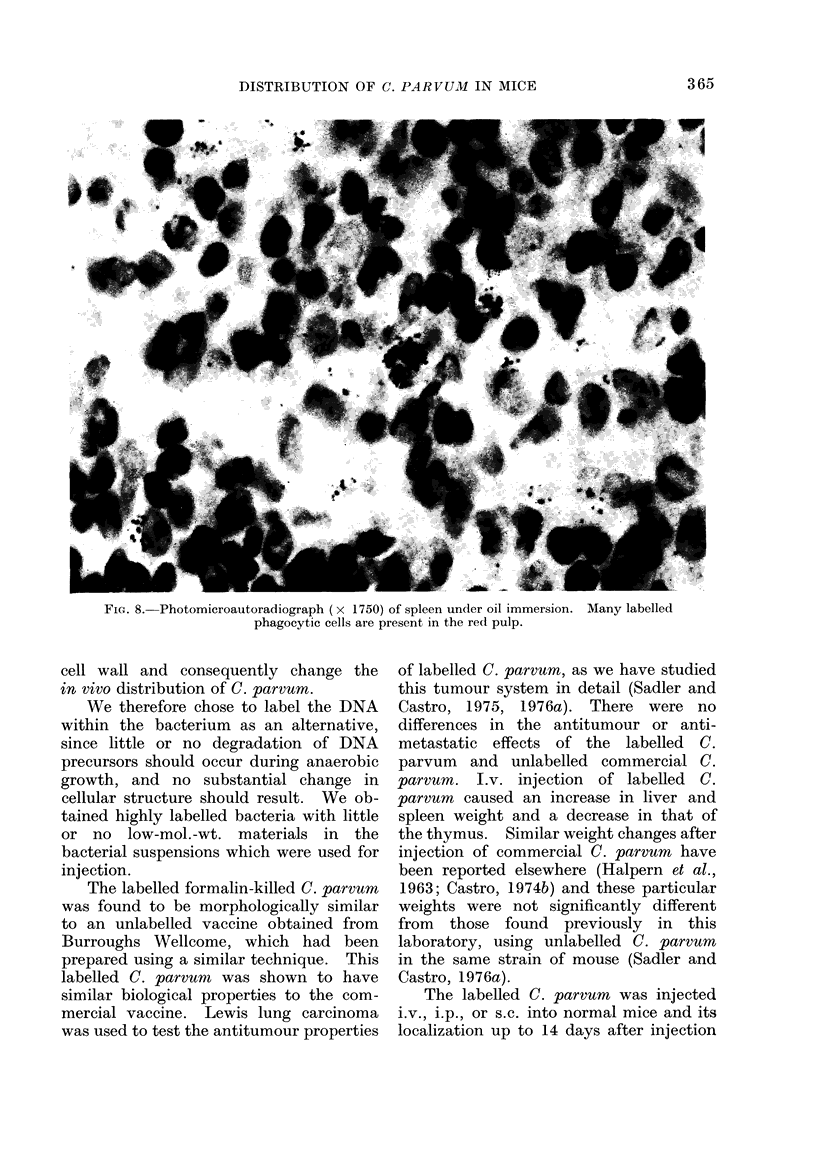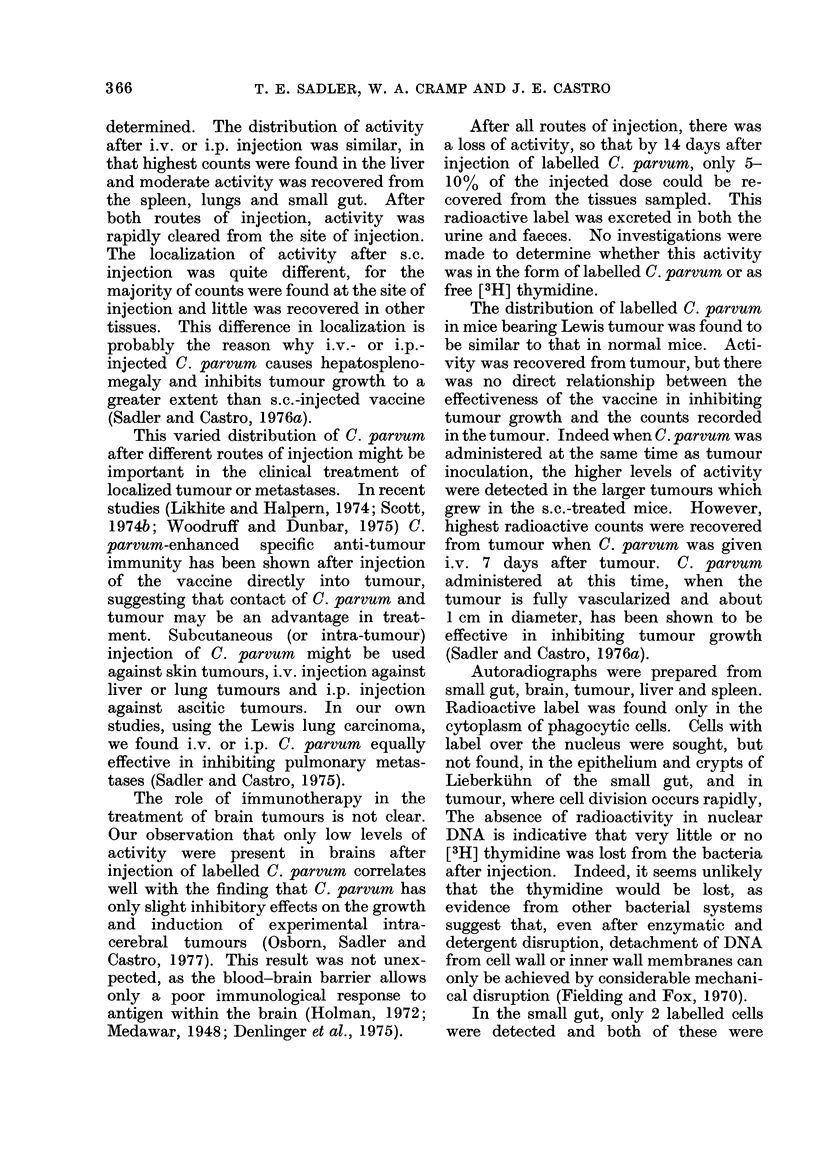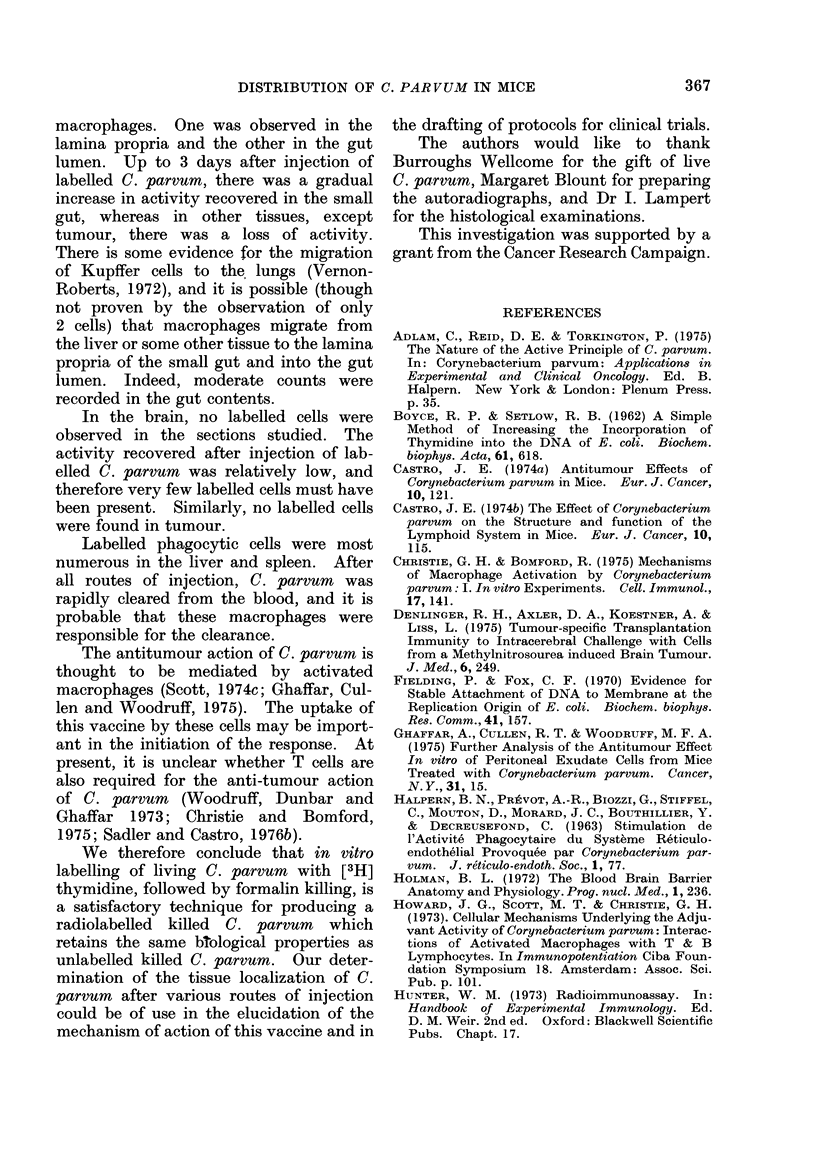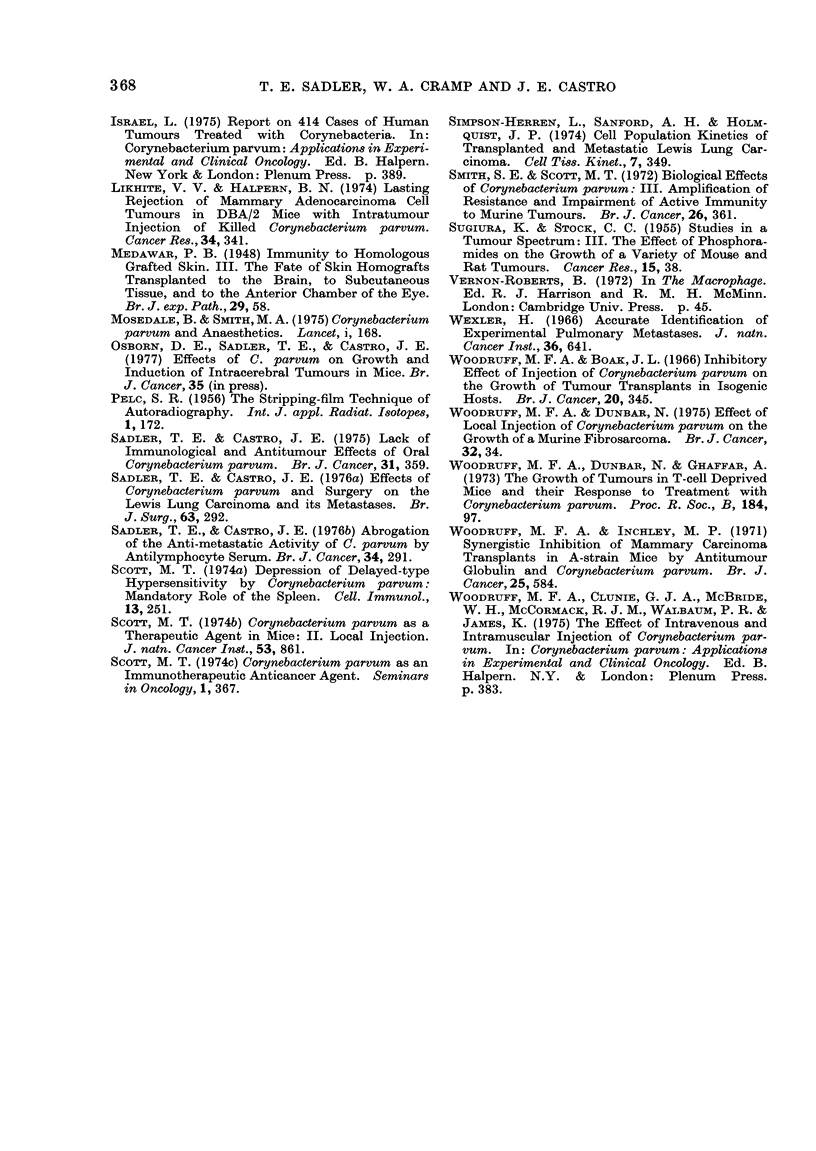# Radiolabelling of Corynebacterium parvum and its distribution in mice.

**DOI:** 10.1038/bjc.1977.50

**Published:** 1977-03

**Authors:** T. E. Sadler, W. A. Cramp, J. E. Castro

## Abstract

**Images:**


*Br. J. Cancer* (1977) **35**, 357.

### RADIOLABELLING OF *CORYNEBACTERIUM PARVUM* AND ITS DISTRIBUTION IN MICE

T. E. SADLER, W. A. CRAMP AND J. E. CASTRO

*From the Urological and Transplantation Unit, Department of Surgery, Royal Postgraduate Medical School and the M.R.C. Cyclotron Unit, Hammersmith Hospital, London W12 0HS*

Received 15 September 1976 Accepted 7 October 1976

**Summary.—*Corynebacterium parvum* was labelled by growing live bacteria in the presence of [$^3$H] thymidine. The bacteria were killed by formalin, washed thoroughly and resuspended at a concentration of 7 mg dry weight/ml. An activity of 1–6 $\times 10^5$ ct/min/0·1 ml was obtained. The biological properties (inhibition of tumour growth and hepatosplenomegaly) of the labelled *C. parvum* were compared with those of commercially available vaccine, and were found to be similar.**

**Labelled *C. parvum* was injected i.v., i.p., or s.c. into normal C57BL mice and the localization of activity determined at 4 h and 1, 3, 7 and 14 days after injection. After i.v. or i.p. injection, highest counts were recorded in the liver. Moderate activity was found in the spleen, lungs and small gut. After s.c. injection, the majority of radioactive label was detected at the site of injection and little found in other tissues. The distribution of injected *C. parvum* was also studied in mice bearing Lewis tumour, and was found to be similar to that in normal mice. Moderate amounts of labelled *C. parvum* were recovered from tumour. There appeared to be no relationship between the antitumour effect of *C. parvum* given by a particular route of injection and the concentration of *C. parvum* recovered from the tumour.**

KILLED *Corynebacterium parvum* has powerful antitumour effects on many syngeneic animal tumours (Woodruff and Boak, 1966; Smith and Scott, 1972; Castro, 1974*a*) and it is now being assessed as an anticancer agent in man (Israel, 1975; Woodruff *et al.*, 1975). This vaccine has been shown in mice to be more effective against tumours if given i.v. or i.p. than if given s.c. (Woodruff and Inchley, 1971; Sadler and Castro, 1975).

*C. parvum* given i.v. stimulates the RES, as judged by clearance of colloidal carbon, and increases spleen and liver size (Halpern *et al.*, 1964; Castro, 1974*b*). It has adjuvant properties (Howard, Scott and Christie, 1973) but in defined circumstances appears to depress cell-mediated immunity (Scott, 1974*a*; Castro, 1974*b*).

Little is known of the localization of *C. parvum in vivo* after injection. It was considered that such information might lead to a better understanding of the biological activity of this agent. Therefore, the aim of this study was to develop a method for radiolabelling *C. parvum*, to study the biological properties of this labelled vaccine, and to observe its distribution in normal and tumour-bearing mice.

### MATERIALS AND METHODS

C. parvum.—Freeze-dried live *C. parvum* (Wellcome, strain CN 6134) was used in the labelling studies. Formalin-killed *C. parvum* (Wellcome, strain CN 6134, batch PX 416, 7 mg dry weight/ml) was used as a control.

*Radioactive labelling*.—An inoculum of the freeze-dried *C. parvum* was added to a 20 ml suspension of Difco Bacto "cooked meat medium" containing 5% foetal calf serum (Gibco Biocult) and 1% glucose. After 7 days at 37°C in a tightly sealed universal bottle, the slurry of meat granules and bacteria was shaken, and 2 ml of the upper layer containing mostly bacteria was added to 100 ml of Difco Bacto " antibiotic medium 3 " containing 1% glucose. After 7 days' growth without aeration, an 8-ml inoculum was taken from this culture and added to 1 l fresh medium (antibiotic medium 3 plus 1% glucose). Deoxyadenosine 0·2 mg/ml (Sigma) was added (Boyce and Setlow, 1962), followed by 1 $\mu$Ci of [$^3$H] thymidine (Radiochemical Centre, Amersham, sp. act. 59 Ci/mmol) after the deoxyadenosine had dissolved. The bacteria were harvested 5 days later, washed $\times$ 3 by centrifugation with 0·9% NaCl, and finally resuspended in normal saline containing 0·5% formalin for 7 days at 37°C. The killed cells were washed free of formalin and resuspended in normal saline containing 0·01% thiomersalate (BDH) as a preservative. The final suspension was concentrated at 7 mg dry weight/ml. The activity of 3 batches of labelled *C. parvum* prepared at different times was between 1 and 6 $\times$ $10^5$ ct/min/0·1 ml.

A comparison of the activity of washed intact killed bacteria and that of the precipitate after disruption with cold 10% TCA showed that all the activity in the bacteria was in the form of high-mol.-wt. macromolecules. There was no loss of label from *C. parvum* during storage. Less than 0.25% of the activity present in the labelled bacteria was found in the supernatant when the vaccine was centrifuged 3 months after labelling. Microscopic examination of the killed labelled *C. parvum* indicated that there was no contamination by other bacteria, and both this radiolabelled *C. parvum* and killed but unlabelled bacteria (prepared using similar techniques by Burroughs Wellcome) were morphologically similar.

*Mice.*—Age-matched adult female C57BL/10 Sc Sn mice obtained from Olac (Southern) Ltd were used.

*Tumour.*—Lewis lung carcinoma was implanted s.c. as a 0·1 ml homogenate in the lower flank. It is an epidermoid tumour which originated spontaneously as a carcinoma of the lung of a female C57BL mouse at the Wistar Institute in 1951 (Sugiura and Stock, 1955). If grown s.c., it always metastasizes to the lungs (Simpson-Herren, Sanford and Holmquist, 1974). For each animal, 2 diameters of the primary tumour were measured twice weekly and the mean diameter calculated. Macroscopic surface lung metastases were counted 21 days after tumour implantation, after staining the lungs by inflation with a dilute solution of Indian ink and fixation in Fekete's solution (Wexler, 1966). The number of metastases in the different experimental groups was compared by Student's *t* test.

C. parvum *injections.*—*C. parvum* was given either i.v. as a dose of 0·44 mg diluted to 0·2 ml in normal saline, or i.p. or s.c. as 0·7 mg in 0·1 ml to untreated mice or to mice immediately after implantation of tumour.

*Distribution studies.*—Groups of 5 mice were anaesthetized with ether and exsanguinated from the retro-orbital sinus at 4 h and 1, 3, 7 and 14 days after injection of labelled *C. parvum*. Peritoneal cells were collected after injecting 3 ml of heparinized saline, i.p., and aspirating the cell-containing fluid after abdominal massage. The following tissues were removed for study: liver, spleen, mesenteric lymph nodes, small gut, kidney, thymus, lungs, heart, brain, skin, muscle and femoral bone marrow. In tumour-bearing mice, the tumour and draining lymph nodes were also removed. Tissues were dissected free of connective tissue, rinsed in saline, blotted dry and weighed. In a separate experiment, bladder urine, faeces and gut contents were collected from groups of 3 mice. Whole organs, or samples of tissues up to 200 mg in weight, were added to scintillation vials containing 1 ml of the tissue solvent, Soluene—350 (Packard); when there was sufficient tissue, samples were studied in triplicate. 10 ml of scintillant (500 ml Triton $\times$ 100, 1 l toluene and 6 g PPO) was added to the dissolved tissues, and the samples counted on an " Intertechnique " liquid scintillation spectrometer. An internal standard was used to correct for quench.

*Calculations.*—The radioactivity of each *whole* organ was determined and expressed as a percentage of the total injected dose. The total radioactivity in the blood was taken to be that in a 1·5 ml volume. Mean values and standard deviations were calculated for all experimental groups.

*Autoradiography.*—Liver, spleen, small gut and brain were excised 3 days after i.v. or i.p. injection of labelled *C. parvum*. Tumour was studied 7 and 14 days after i.v. injection of the vaccine. The tissues were fixed in formol saline and autoradiographs were prepared using the standard stripping-film technique (Pelc, 1956). Histological sections were stained with haematoxylin and eosin.

### RESULTS

### *Some biological properties of labelled* Corynebacterium parvum

*Antitumour action.*—Fig. 1 shows the effect of labelled and control *C. parvum* on the growth of the primary Lewis

FIG. 1.—The effect of [$^3$H] thymidine-labelled *C. parvum* given either i.v. ● --- ●, or i.p. ■——■, and control *C. parvum* i.v. ○ --- ○, or i.p. □——□ on the growth of the primary Lewis lung carcinoma. Control mice were given saline ×——×. Each point represents the mean of 6–8 mice and bar denotes s.e.

tumour when injected either i.v. or i.p. at the same time as tumour into groups of 8 mice. *C. parvum* caused a significant inhibition of tumour growth when compared with that in control mice given saline (on Day 21, $P = 0{\cdot}001$). There was no significant difference in tumour growth between the 2 forms of *C. parvum* after similar routes of injection.

The numbers of pulmonary metastases found 21 days after tumour implantation are shown in the Table. There was significant reduction ($P < 0{\cdot}001$) of metastases in all mice which received *C. parvum*. But there was no difference in the number of metastases between the different *C. parvum* groups.

*Hepatosplenomegaly.*—Groups of 5 mice were injected i.v. with labelled *C. parvum* and killed 4 h and 1, 3, 7 and 14 days after injection. The whole body, liver, spleen and thymus were weighed at each interval after injection (Fig. 2.). There was an increase in the weight of the liver and spleen, and a decrease in that of the thymus. There was a slight drop in body weight, which had returned to normal by 14 days after *C. parvum*.

### *Distribution of* C. parvum *in normal mice*

*Intravenous injection.*—The distribution of activity after i.v. injection of [$^3$H) thymidine-labelled *C. parvum* is shown in Fig. 3. Activity was rapidly cleared from the blood: only 0·2% of the injected dose could be recovered 4 h after injection. This value rose to 0·75% on Days 1 and 3, and subsequently fell. Only moderate counts were detected in the lungs: 4% of the injected dose was present at 4 h and this value thereafter decreased. Highest activity was found in the liver: 4 h after injection the liver contained 59% of the injected dose, by 1 and 3 days it was 36% and at 7 days it had fallen to 20%. By 14 days only 10% was detected. Moderate amounts of radioactive label

TABLE—*The Effect of [$^3$H] Thymidine-labelled and Control* C. parvum *on Metastases from the Lewis Lung Tumour*

| | | No. of mice | Mean metastases ± s.d. |
|---|---|---|---|
| a. | Saline | 7 | 36±9 |
| b. | Control *C. parvum* i.v. | 8 | 8±6 |
| c. | [$^3$H] thymidine *C. parvum* i.v. | 6 | 5±3 |
| d. | Control *C. parvum* i.p. | 8 | 7±5 |
| e. | [$^3$H] thymidine *C. parvum* i.p. | 6 | 7±5 |

Significance by Student's *t* test: a: b, c, d, or e <0·001; b: c, d, e not significant.

FIG. 2.—Liver, spleen, thymus and whole body weights at intervals after i.v. injection of [$^3$H] thymidine-labelled *C. parvum*. Each figure is the mean from 5 mice and bar denotes s.d.

were recorded in the spleen (2·5% at 4 h, 3% at 1 day and 1–1·5% at later times) and the small gut (1·5% at 4 h, 6% at 1 day and progressively less thereafter). In the peritoneal fluid and brain, only slight activity was detected, and no consistently significant levels of radioactivity were recovered from the mesenteric lymph nodes, thymus, heart, bone marrow, skin or muscle.

*Intraperitoneal injection.*—The distribution of radioactivity after i.p. injection of labelled *C. parvum* is shown in Fig. 4. Activity was rapidly cleared from the peritoneal cavity. At 4 h after injection only 8% of the injected dose was recorded. This value had decreased to 4% at 1 day, and there was a further reduction to 3% at 3 days and to 1% at 7 days. Highest activity was found in the liver. At 4 h, 26% was detected. This value gradually decreased, until only 4% was present at 14 days. Moderate amounts of radioactive label were recovered from the spleen (4% at 4 h, thereafter decreasing) and the small gut (2·5% at 4 h, gradually increasing to a maximum of 5% at 3 days). A moderate count (3·5%) was recorded in the lungs at 4 h, but little was found at later times. Only slight activity was found in the brain and blood, and no consistently significant activity was recovered from the mesenteric lymph nodes, thymus, heart, bone marrow, skin or muscle.

*Subcutaneous injection.*—The distribution of activity after s.c. injection of radiolabelled *C. parvum* is shown in Fig. 5. After this route of injection, highest activity was recovered from the site of injection. Seventy-one per cent of the injected dose was detected at 4 h after injection, and a similar number of counts were found at Day 1. However, by 3

FIG. 3.—Distribution of activity recovered as % of total injected dose, in various tissues at 4 h, and 1, 3, 7 and 14 days after i.v. injection of [$^3$H] thymidine-labelled *C. parvum* in normal mice. Each histogram block represents the mean value from 5 animals and bar denotes s.d.

days the value had diminished to 37%, and by Day 14 only 2% was recovered. Very little radioactive label was detected in the lymph nodes draining the site of injection, just 0·1% on Days 3 and 7. Moderate counts were recovered from the liver and small gut, both increasing to a maximum on Day 3, of 2% and 4% respectively. In the spleen, peritoneal fluid, brain and blood, only slight radioactivity was detected, and no consistently significant level of activity was found in the lungs, mesenteric lymph nodes, thymus, heart, bone marrow, skin or muscle.

*Loss of radioactivity*.—After all routes of injection, there was a loss of activity (Fig. 3, 4 and 5). At 14 days after injection, only 5–10% of the injected dose was recovered. There was no difference in the activities detected in the kidney, urine, faeces and gut contents after i.v., i.p. or s.c. injection. However, the values did vary from mouse to mouse. Between 0·2% and 1·0% of the injected dose was found in the kidney at all times after injection. Up to 5% was found in 0·1 ml urine and 2–3% in the content of both the small and large bowel at 4 h, and 1 and 3 days after injection. Less activity was recorded at 7 and 14 days.

*Distribution of* C. parvum *in tumour-bearing mice*

Radiolabelled *C. parvum* was injected i.v., i.p., or s.c. on the same day as the mice were inoculated s.c. with Lewis lung carcinoma. The distribution of radioactivity in these tumour-bearing mice was found to be similar to that in normal mice (Figs. 3, 4 and 5). The radioactive label detected in tumour after the different routes of injection is shown in Fig. 6a. There was an increase in activity, which correlated to some extent with tumour size. Up to 3 days after injection, tumour weights varied between 90 and 110 mg,

FIG. 4.—Distribution of activity recovered as % of total injected dose, in various tissues at 4 h and, 1, 3, 7 and 14 days after i.p. injection of [$^3$H] thymidine-labelled *C. parvum* in normal mice. Each histogram block represents the mean value from 5 animals and bar denotes s.d.

and the counts recorded between 0·1 and 0·2% of the injected dose. By 7 days after i.v. injection, tumour weight had increased to 275 mg, and the activity recovered to 1·3%. However, by 14 days, when tumour weight was 1202 mg, only 1·4% was detected. After i.p. injection, 0·9% of the injected dose was found in tumours weighing 181 and 1365 mg, at 7 and 14 days after injection, respectively. Seven days after s.c. injection, 1·0% was found in tumours weighing 520 mg. This value rose to 2·3% by 14 days, when tumour weighed 2145 mg. No significant activity was recovered from the lymph nodes draining these tumours.

A higher level of activity was found in tumour when *C. parvum* was injected i.v. 7 days after tumour inoculation (Fig. 6b). 2·9% of the injected dose was detected at 1 day, when tumour weighed 453 mg. By 14 days the activity recovered had risen to 5·6% and tumour weight to 1714 mg. 0·1% of the injected dose was recorded in the lymph nodes draining these tumours.

*Autoradiography*

Autoradiographs were prepared from liver, spleen, small gut and brain taken from mice 3 days after i.v. or i.p. injection of labelled *C. parvum*. Tumour was studied either 14 days after i.v. injection of *C. parvum* from mice given the vaccine at the same time as tumour inoculation, or 7 days after injection when the vaccine was given 7 days after tumour.

*Liver*.—The liver was heavily labelled (Fig. 7). Grains were observed in the cytoplasm of Kuppfer cells and in histiocytes around the periphery of granulomas. (Systemic *C. parvum* is known to cause

FIG. 5.—Distribution of activity recovered as % of total injected dose, in various tissues at 4 h, and 1, 3, 7 and 14 days after s.c. injection of [$^3$H] thymidine-labelled *C. parvum*. Each histogram block represents the mean value from 5 animals and bar denotes s.d.

FIG. 6.—Distribution of activity recovered as % of total injected dose, in tumour at 4 h, and 1, 3, 7 and 14 days after (a) i.v., i.p., or s.c. injection of *C. parvum* given at the same time as tumour inoculation, and (b) i.v. *C. parvum* given 7 days after tumour.

FIG. 7.—Photomicroautoradiograph (× 1750) of liver, under oil immersion. In the centre of the picture are labelled Kupffer cells. Part of a granulomatous lesion is present lower right.

granuloma formation in the liver (Castro, 1974*b*; Mosedale and Smith, 1975).)

*Spleen*.—Grains were observed in the phagocytic cells of the spleen (Fig. 8). These were mainly present in the red pulp, in the sinus lining cells and the chords. A few labelled cells were found in the white pulp near the marginal sinus.

*Small gut*.—Only 2 labelled cells were found in the small gut sections and both of these were phagocytic. One was observed in the lamina propria and the other in the gut lumen. No label was found in gut epithelium or crypts of Lieberkühn.

*Brain*.—There were no labelled cells in the sections of brain which were examined.

*Tumour*.—Many dividing tumour cells were seen. No labelled cells were observed in the sections which were examined.

### DISCUSSION

An ideal radioactive marker for the determination of the location of *C. parvum* after injection would be labelled cell wall or inner wall membrane, since it is these constituents which stimulate the reticuloendothelial system (Adlam, Reid & Torkington, 1975). However, the available nutrients for the incorporation of radiolabel into these sites are the $^{14}C$- or $^{3}H$-labelled sugars and glycerol, and these substances are extensively degraded to $CO_2$ during growth of the bacteria under anaerobic conditions. Another method of labelling the cell wall would be to iodinate a killed bacterial suspension, using Chloramine T and $^{125}I$ (Hunter, 1973). However, we considered that this technique, which substitutes $^{125}I$ into the surface tyrosine groups, might alter the bacterial

FIG. 8.—Photomicroautoradiograph (× 1750) of spleen under oil immersion. Many labelled phagocytic cells are present in the red pulp.

cell wall and consequently change the *in vivo* distribution of *C. parvum*.

We therefore chose to label the DNA within the bacterium as an alternative, since little or no degradation of DNA precursors should occur during anaerobic growth, and no substantial change in cellular structure should result. We obtained highly labelled bacteria with little or no low-mol.-wt. materials in the bacterial suspensions which were used for injection.

The labelled formalin-killed *C. parvum* was found to be morphologically similar to an unlabelled vaccine obtained from Burroughs Wellcome, which had been prepared using a similar technique. This labelled *C. parvum* was shown to have similar biological properties to the commercial vaccine. Lewis lung carcinoma was used to test the antitumour properties of labelled *C. parvum*, as we have studied this tumour system in detail (Sadler and Castro, 1975, 1976*a*). There were no differences in the antitumour or antimetastatic effects of the labelled *C.* parvum and unlabelled commercial *C. parvum*. I.v. injection of labelled *C. parvum* caused an increase in liver and spleen weight and a decrease in that of the thymus. Similar weight changes after injection of commercial *C. parvum* have been reported elsewhere (Halpern *et al.*, 1963; Castro, 1974*b*) and these particular weights were not significantly different from those found previously in this laboratory, using unlabelled *C. parvum* in the same strain of mouse (Sadler and Castro, 1976*a*).

The labelled *C. parvum* was injected i.v., i.p., or s.c. into normal mice and its localization up to 14 days after injection determined. The distribution of activity after i.v. or i.p. injection was similar, in that highest counts were found in the liver and moderate activity was recovered from the spleen, lungs and small gut. After both routes of injection, activity was rapidly cleared from the site of injection. The localization of activity after s.c. injection was quite different, for the majority of counts were found at the site of injection and little was recovered in other tissues. This difference in localization is probably the reason why i.v.- or i.p.-injected *C. parvum* causes hepatosplenomegaly and inhibits tumour growth to a greater extent than s.c.-injected vaccine (Sadler and Castro, 1976*a*).

This varied distribution of *C. parvum* after different routes of injection might be important in the clinical treatment of localized tumour or metastases. In recent studies (Likhite and Halpern, 1974; Scott, 1974*b*; Woodruff and Dunbar, 1975) *C. parvum*-enhanced specific anti-tumour immunity has been shown after injection of the vaccine directly into tumour, suggesting that contact of *C. parvum* and tumour may be an advantage in treatment. Subcutaneous (or intra-tumour) injection of *C. parvum* might be used against skin tumours, i.v. injection against liver or lung tumours and i.p. injection against ascitic tumours. In our own studies, using the Lewis lung carcinoma, we found i.v. or i.p. *C. parvum* equally effective in inhibiting pulmonary metastases (Sadler and Castro, 1975).

The role of immunotherapy in the treatment of brain tumours is not clear. Our observation that only low levels of activity were present in brains after injection of labelled *C. parvum* correlates well with the finding that *C. parvum* has only slight inhibitory effects on the growth and induction of experimental intracerebral tumours (Osborn, Sadler and Castro, 1977). This result was not unexpected, as the blood–brain barrier allows only a poor immunological response to antigen within the brain (Holman, 1972; Medawar, 1948; Denlinger *et al.*, 1975).

After all routes of injection, there was a loss of activity, so that by 14 days after injection of labelled *C. parvum*, only 5–10% of the injected dose could be recovered from the tissues sampled. This radioactive label was excreted in both the urine and faeces. No investigations were made to determine whether this activity was in the form of labelled *C. parvum* or as free [$^3$H] thymidine.

The distribution of labelled *C. parvum* in mice bearing Lewis tumour was found to be similar to that in normal mice. Activity was recovered from tumour, but there was no direct relationship between the effectiveness of the vaccine in inhibiting tumour growth and the counts recorded in the tumour. Indeed when *C. parvum* was administered at the same time as tumour inoculation, the higher levels of activity were detected in the larger tumours which grew in the s.c.-treated mice. However, highest radioactive counts were recovered from tumour when *C. parvum* was given i.v. 7 days after tumour. *C. parvum* administered at this time, when the tumour is fully vascularized and about 1 cm in diameter, has been shown to be effective in inhibiting tumour growth (Sadler and Castro, 1976*a*).

Autoradiographs were prepared from small gut, brain, tumour, liver and spleen. Radioactive label was found only in the cytoplasm of phagocytic cells. Cells with label over the nucleus were sought, but not found, in the epithelium and crypts of Lieberkühn of the small gut, and in tumour, where cell division occurs rapidly, The absence of radioactivity in nuclear DNA is indicative that very little or no [$^3$H] thymidine was lost from the bacteria after injection. Indeed, it seems unlikely that the thymidine would be lost, as evidence from other bacterial systems suggest that, even after enzymatic and detergent disruption, detachment of DNA from cell wall or inner wall membranes can only be achieved by considerable mechanical disruption (Fielding and Fox, 1970).

In the small gut, only 2 labelled cells were detected and both of these were macrophages. One was observed in the lamina propria and the other in the gut lumen. Up to 3 days after injection of labelled *C. parvum*, there was a gradual increase in activity recovered in the small gut, whereas in other tissues, except tumour, there was a loss of activity. There is some evidence for the migration of Kupffer cells to the lungs (Vernon-Roberts, 1972), and it is possible (though not proven by the observation of only 2 cells) that macrophages migrate from the liver or some other tissue to the lamina propria of the small gut and into the gut lumen. Indeed, moderate counts were recorded in the gut contents.

In the brain, no labelled cells were observed in the sections studied. The activity recovered after injection of labelled *C. parvum* was relatively low, and therefore very few labelled cells must have been present. Similarly, no labelled cells were found in tumour.

Labelled phagocytic cells were most numerous in the liver and spleen. After all routes of injection, *C. parvum* was rapidly cleared from the blood, and it is probable that these macrophages were responsible for the clearance.

The antitumour action of *C. parvum* is thought to be mediated by activated macrophages (Scott, 1974*c*; Ghaffar, Cullen and Woodruff, 1975). The uptake of this vaccine by these cells may be important in the initiation of the response. At present, it is unclear whether T cells are also required for the anti-tumour action of *C. parvum* (Woodruff, Dunbar and Ghaffar 1973; Christie and Bomford, 1975; Sadler and Castro, 1976*b*).

We therefore conclude that *in vitro* labelling of living *C. parvum* with [$^3$H] thymidine, followed by formalin killing, is a satisfactory technique for producing a radiolabelled killed *C. parvum* which retains the same biological properties as unlabelled killed *C. parvum*. Our determination of the tissue localization of *C. parvum* after various routes of injection could be of use in the elucidation of the mechanism of action of this vaccine and in the drafting of protocols for clinical trials.

The authors would like to thank Burroughs Wellcome for the gift of live *C. parvum*, Margaret Blount for preparing the autoradiographs, and Dr I. Lampert for the histological examinations.

This investigation was supported by a grant from the Cancer Research Campaign.

### REFERENCES

ADLAM, C., REID, D. E. & TORKINGTON, P. (1975) The Nature of the Active Principle of *C. parvum*. In: Corynebacterium parvum: *Applications in Experimental and Clinical Oncology*. Ed. B. Halpern. New York & London: Plenum Press. p. 35.

BOYCE, R. P. & SETLOW, R. B. (1962) A Simple Method of Increasing the Incorporation of Thymidine into the DNA of *E. coli*. *Biochem. biophys. Acta*, **61**, 618.

CASTRO, J. E. (1974*a*) Antitumour Effects of *Corynebacterium parvum* in Mice. *Eur. J. Cancer*, **10**, 121.

CASTRO, J. E. (1974*b*) The Effect of *Corynebacterium parvum* on the Structure and function of the Lymphoid System in Mice. *Eur. J. Cancer*, **10**, 115.

CHRISTIE, G. H. & BOMFORD, R. (1975) Mechanisms of Macrophage Activation by *Corynebacterium parvum*: I. *In vitro* Experiments. *Cell. Immunol.*, **17**, 141.

DENLINGER, R. H., AXLER, D. A., KOESTNER, A. & LISS, L. (1975) Tumour-specific Transplantation Immunity to Intracerebral Challenge with Cells from a Methylnitrosourea induced Brain Tumour. *J. Med.*, **6**, 249.

FIELDING, P. & FOX, C. F. (1970) Evidence for Stable Attachment of DNA to Membrane at the Replication Origin of *E. coli*. *Biochem. biophys. Res. Comm.*, **41**, 157.

GHAFFAR, A., CULLEN, R. T. & WOODRUFF, M. F. A. (1975) Further Analysis of the Antitumour Effect *In vitro* of Peritoneal Exudate Cells from Mice Treated with *Corynebacterium parvum*. *Cancer, N.Y.*, **31**, 15.

HALPERN, B. N., PRÉVOT, A.-R., BIOZZI, G., STIFFEL, C., MOUTON, D., MORARD, J. C., BOUTHILLIER, Y. & DECREUSEFOND, C. (1963) Stimulation de l'Activité Phagocytaire du Système Réticulo-endothélial Provoquée par *Corynebacterium parvum*. *J. réticulo-endoth. Soc.*, **1**, 77.

HOLMAN, B. L. (1972) The Blood Brain Barrier Anatomy and Physiology. *Prog. nucl. Med.*, **1**, 236.

HOWARD, J. G., SCOTT, M. T. & CHRISTIE, G. H. (1973). Cellular Mechanisms Underlying the Adjuvant Activity of *Corynebacterium parvum*: Interactions of Activated Macrophages with T & B Lymphocytes. In *Immunopotentiation* Ciba Foundation Symposium 18. Amsterdam: Assoc. Sci. Pub. p. 101.

HUNTER, W. M. (1973) Radioimmunoassay. In: *Handbook of Experimental Immunology*. Ed. D. M. Weir. 2nd ed. Oxford: Blackwell Scientific Pubs. Chapt. 17.

ISRAEL, L. (1975) Report on 414 Cases of Human Tumours Treated with Corynebacteria. In: Corynebacterium parvum: *Applications in Experimental and Clinical Oncology*. Ed. B. Halpern. New York & London: Plenum Press. p. 389.

LIKHITE, V. V. & HALPERN, B. N. (1974) Lasting Rejection of Mammary Adenocarcinoma Cell Tumours in DBA/2 Mice with Intratumour Injection of Killed *Corynebacterium parvum*. *Cancer Res.*, **34**, 341.

MEDAWAR, P. B. (1948) Immunity to Homologous Grafted Skin. III. The Fate of Skin Homografts Transplanted to the Brain, to Subcutaneous Tissue, and to the Anterior Chamber of the Eye. *Br. J. exp. Path.*, **29**, 58.

MOSEDALE, B. & SMITH, M. A. (1975) *Corynebacterium parvum* and Anaesthetics. *Lancet*, i, 168.

OSBORN, D. E., SADLER, T. E., & CASTRO, J. E. (1977) Effects of *C. parvum* on Growth and Induction of Intracerebral Tumours in Mice. *Br. J. Cancer*, **35** (in press).

PELC, S. R. (1956) The Stripping-film Technique of Autoradiography. *Int. J. appl. Radiat. Isotopes*, **1**, 172.

SADLER, T. E. & CASTRO, J. E. (1975) Lack of Immunological and Antitumour Effects of Oral *Corynebacterium parvum*. *Br. J. Cancer*, **31**, 359.

SADLER, T. E. & CASTRO, J. E. (1976*a*) Effects of *Corynebacterium parvum* and Surgery on the Lewis Lung Carcinoma and its Metastases. *Br. J. Surg.*, **63**, 292.

SADLER, T. E., & CASTRO, J. E. (1976*b*) Abrogation of the Anti-metastatic Activity of *C. parvum* by Antilymphocyte Serum. *Br. J. Cancer*, **34**, 291.

SCOTT, M. T. (1974*a*) Depression of Delayed-type Hypersensitivity by *Corynebacterium parvum*: Mandatory Role of the Spleen. *Cell. Immunol.*, **13**, 251.

SCOTT, M. T. (1974*b*) *Corynebacterium parvum* as a Therapeutic Agent in Mice: II. Local Injection. *J. natn. Cancer Inst.*, **53**, 861.

SCOTT, M. T. (1974*c*) *Corynebacterium parvum* as an Immunotherapeutic Anticancer Agent. *Seminars in Oncology*, **1**, 367.

SIMPSON-HERREN, L., SANFORD, A. H. & HOLMQUIST, J. P. (1974) Cell Population Kinetics of Transplanted and Metastatic Lewis Lung Carcinoma. *Cell Tiss. Kinet.*, **7**, 349.

SMITH, S. E. & SCOTT, M. T. (1972) Biological Effects of *Corynebacterium parvum*: III. Amplification of Resistance and Impairment of Active Immunity to Murine Tumours. *Br. J. Cancer*, **26**, 361.

SUGIURA, K. & STOCK, C. C. (1955) Studies in a Tumour Spectrum: III. The Effect of Phosphoramides on the Growth of a Variety of Mouse and Rat Tumours. *Cancer Res.*, **15**, 38.

VERNON-ROBERTS, B. (1972) In *The Macrophage*. Ed. R. J. Harrison and R. M. H. McMinn. London: Cambridge Univ. Press. p. 45.

WEXLER, H. (1966) Accurate Identification of Experimental Pulmonary Metastases. *J. natn. Cancer Inst.*, **36**, 641.

WOODRUFF, M. F. A. & BOAK, J. L. (1966) Inhibitory Effect of Injection of *Corynebacterium parvum* on the Growth of Tumour Transplants in Isogenic Hosts. *Br. J. Cancer*, **20**, 345.

WOODRUFF, M. F. A. & DUNBAR, N. (1975) Effect of Local Injection of *Corynebacterium parvum* on the Growth of a Murine Fibrosarcoma. *Br. J. Cancer*, **32**, 34.

WOODRUFF, M. F. A., DUNBAR, N. & GHAFFAR, A. (1973) The Growth of Tumours in T-cell Deprived Mice and their Response to Treatment with *Corynebacterium parvum*. *Proc. R. Soc., B*, **184**, 97.

WOODRUFF, M. F. A. & INCHLEY, M. P. (1971) Synergistic Inhibition of Mammary Carcinoma Transplants in A-strain Mice by Antitumour Globulin and *Corynebacterium parvum*. *Br. J. Cancer*, **25**, 584.

WOODRUFF, M. F. A., CLUNIE, G. J. A., MCBRIDE, W. H., MCCORMACK, R. J. M., WALBAUM, P. R. & JAMES, K. (1975) The Effect of Intravenous and Intramuscular Injection of *Corynebacterium parvum*. In: *Corynebacterium parvum: Applications in Experimental and Clinical Oncology*. Ed. B. Halpern. N.Y. & London: Plenum Press. p. 383.